# Supplementary material for: Mapping the evolution of fertility support policies in China: A content and instrumental analysis
Source: PLoS One. 2025 Oct 9;20(10):e0332137. doi: 10.1371/journal.pone.0332137 (PMC12510515; doi:10.1371/journal.pone.0332137)
Supplement: S1 Appendix — (ZIP) [file pone.0332137.s001.zip › S1 Appendix. 226 original policy documents/35-国家卫生健康委关于印发托育机构保育指导大纲(试行)的通知(FBM-CLI-4-351300).doc]

国家卫生健康委关于印发托育机构保育指导大纲(试行)的通知

**发布部门：** [国家卫生健康委员会](https://www.pkulaw.com/law/chl?Aggs.IssueDepartment=60321) [机构沿革](javascript:void(0))

**发文字号：**国卫人口发〔2021〕2号

**发布日期：**2021.01.12

**实施日期：**2021.01.12

**时效性：** [现行有效](https://www.pkulaw.com/law/chl?Aggs.TimelinessDic=01)

**效力级别：** [部门规范性文件](https://www.pkulaw.com/law/chl?Aggs.EffectivenessDic=XE0302)

**法规类别：** [卫生综合规定](https://www.pkulaw.com/law/chl?Aggs.Category=09601)

国家卫生健康委关于印发托育机构保育指导大纲（试行）的通知
（国卫人口发〔2021〕2号）

各省、自治区、直辖市及新疆生产建设兵团卫生健康委：
　　为指导托育机构为3岁以下婴幼儿提供科学、规范的照护服务，按照《[国务院办公厅关于促进3岁以下婴幼儿照护服务发展的指导意见](https://www.pkulaw.com/chl/434b24b8b5769383bdfb.html)》（国办发〔2019〕15号）的要求，我委组织制定了《托育机构保育指导大纲（试行）》（可从国家卫生健康委网站下载）。现予印发，请遵照执行、推动落实。

国家卫生健康委
2021年1月12日

托育机构保育指导大纲（试行）

　第一章　总则

　　一、为贯彻《[国务院办公厅关于促进3岁以下婴幼儿照护服务发展的指导意见](https://www.pkulaw.com/chl/434b24b8b5769383bdfb.html)》，依据国家卫生健康委《托育机构设置标准（试行）》《托育机构管理规范（试行）》，指导托育机构为3岁以下婴幼儿（以下简称婴幼儿）提供科学、规范的照护服务，促进婴幼儿健康成长，特制定本大纲。

　　二、本大纲适用于经有关部门登记、卫生健康部门备案，为婴幼儿提供全日托、半日托等照护服务的托育机构。提供计时托、临时托等照护服务的托育机构可参照执行。

　　三、托育机构保育是婴幼儿照护服务的重要组成部分，是生命全周期服务管理的重要内容。通过创设适宜环境，合理安排一日生活和活动，提供生活照料、安全看护、平衡膳食和早期学习机会，促进婴幼儿身体和心理的全面发展。

　　四、托育机构保育应遵循以下基本原则：
　　（一）尊重儿童。坚持儿童优先，保障儿童权利。尊重婴幼儿成长特点和规律，关注个体差异，促进每个婴幼儿全面发展。
　　（二）安全健康。最大限度地保护婴幼儿的安全和健康，切实做好托育机构的安全防护、营养膳食、疾病防控等工作。
　　（三）积极回应。提供支持性环境，敏感观察婴幼儿，理解其生理和心理需求，并及时给予积极适宜的回应。
　　（四）科学规范。按照国家和地方相关标准和规范，合理安排婴幼儿的生活和活动，满足婴幼儿生长发育的需要。

　第二章　目标与要求

　　托育机构保育工作应当遵循婴幼儿发展的年龄特点与个体差异，通过多种途径促进婴幼儿身体发育和心理发展。保育重点应当包括营养与喂养、睡眠、生活与卫生习惯、动作、语言、认知、情感与社会性等。
　　一、营养与喂养
　　（一）目标。
　　1.获取安全、营养的食物，达到正常生长发育水平；
　　2.养成良好的饮食行为习惯。
　　（二）保育要点。
　　1. 7-12个月
　　（1）继续母乳喂养，不能继续母乳喂养的婴儿使用配方奶喂养。
　　（2）及时添加辅食，从富含铁的泥糊状食物开始，遵循由一种到多种、由少到多、由稀到稠、由细到粗的原则。辅食不添加糖、盐等调味品。
　　（3）每引入新食物要密切观察婴儿是否有皮疹、呕吐、腹泻等不良反应。
　　（4）注意观察婴儿所发出的饥饿或饱足的信号，并及时、恰当回应，不强迫喂食。
　　（5）鼓励婴儿尝试自己进食，培养进餐兴趣。
　　2.　13-24个月
　　（1）继续母乳或配方奶喂养，可以引入奶制品作为辅食，每日提供多种类食物。
　　（2）鼓励和协助幼儿自己进食，关注幼儿以语言、肢体动作等发出进食需求，顺应喂养。
　　（3）培养幼儿使用水杯喝水的习惯，不提供含糖饮料。
　　3.　25-36个月
　　（1）每日提供多种类食物。
　　（2）引导幼儿认识和喜爱食物，培养幼儿专注进食习惯、选择多种食物的能力。
　　（3）鼓励幼儿参与协助分餐、摆放餐具等活动。
　　（三）指导建议。
　　1.制定膳食计划和科学食谱，为婴幼儿提供与年龄发育特点相适应的食物，规律进餐，为有特殊饮食需求的婴幼儿提供喂养建议。
　　2.为婴幼儿创造安静、轻松、愉快的进餐环境，协助婴幼儿进食，并鼓励婴幼儿表达需求、及时回应，顺应喂养，不强迫进食。
　　3.有效控制进餐时间，加强进餐看护，避免发生伤害。

　　二、睡眠
　　（一）目标。
　　1.获得充足睡眠；
　　2.养成独自入睡和作息规律的良好睡眠习惯。
　　（二）保育要点。
　　1.　7-12个月
　　（1）识别婴儿困倦的信号，通过常规睡前活动，培养婴儿独自入睡。
　　（2）帮助婴儿采用仰卧位或侧卧位姿势入睡，脸和头不被遮盖。
　　（3）注意观察婴儿睡眠状态，减少抱睡、摇睡等安抚行为。
　　2.　13-24个月
　　（1）固定幼儿睡眠和唤醒时间，逐渐建立规律的睡眠模式。
　　（2）坚持开展睡前活动，确保幼儿进入较安静状态。
　　（3）培养幼儿独自入睡的习惯。
　　3.　25-36个月
　　（1）规律作息，每日有充足的午睡时间。
　　（2）引导幼儿自主做好睡眠准备，养成良好的睡眠习惯。
　　（三）指导建议。
　　1.为婴幼儿提供良好的睡眠环境和设施，温湿度适宜，白天睡眠不过度遮蔽光线，设立独立床位，保障安全、卫生。
　　2.加强睡眠过程巡视与照护，注意观察婴幼儿睡眠时的面色、呼吸、睡姿，避免发生伤害。
　　3.关注个体差异及睡眠问题，采取适宜的照护方式。

　　三、生活与卫生习惯
　　（一）目标。
　　1.学习盥洗、如厕、穿脱衣服等生活技能；
　　2.逐步养成良好的生活卫生习惯。
　　（二）保育要点。
　　1.　7-12个月
　　（1）及时更换尿布，保持臀部和身体干爽清洁。
　　（2）生活照护过程中，注重与婴儿互动交流。
　　（3）识别及回应婴儿哭闹、四肢活动等表达的需求。
　　2.　13-24个月
　　（1）鼓励幼儿及时表达大小便需求，形成一定的排便规律，逐渐学会自己坐便盆。
　　（2）协助和引导幼儿自己洗手、穿脱衣服等。
　　（3）引导和帮助幼儿学会咳嗽和打喷嚏的方法。
　　3.　25-36个月
　　（1）培养幼儿主动如厕。
　　（2）引导幼儿餐后漱口，使用肥皂或洗手液正确洗手，认识自己的毛巾并擦手。
　　（3）鼓励幼儿自己穿脱衣服。
　　（三）指导建议。
　　1.保持生活场所的安全卫生，预防异物吸入、烧烫伤、跌落伤、溺水、中毒等伤害发生。
　　2.在生活中逐渐养成婴幼儿良好习惯，做好回应性照护，引导其逐步形成规则和安全意识。
　　3.注意培养婴幼儿良好的用眼习惯，限制屏幕时间。
　　4.注意培养婴幼儿良好的口腔卫生习惯，预防龋齿。
　　5.在各生活环节中，做好观察，发现有精神状态不良、烦躁、咳嗽、打喷嚏、呕吐等表现的婴幼儿，要加强看护，必要时及时隔离，并联系家长。

　　四、动作
　　（一）目标。
　　1.掌握基本的大运动技能；
　　2.达到良好的精细动作发育水平。
　　（二）保育要点。
　　1. 7-12个月
　　（1）鼓励婴儿进行身体活动，尤其是地板上的游戏活动。
　　（2）鼓励婴儿自主探索从躺位变成坐位，从坐位转为爬行，逐渐到扶站、扶走。
　　（3）提供适宜的玩具，促进抓、捏、握等精细动作发育。
　　2. 13-24个月
　　（1）鼓励幼儿进行形式多样的身体活动，为幼儿提供参加爬、走、跑、钻、踢、跳等活动的机会。
　　（2）提供多种类活动材料，促进涂画、拼搭、叠套等精细动作发育。
　　（3）鼓励幼儿自己喝水、用小勺吃饭、自己翻书等。
　　3.　25-36个月
　　（1）为幼儿提供参加走直线、跑、跨越低矮障碍物、双脚跳、单足站立、原地单脚跳、上下楼梯等活动的机会。
　　（2）提供多种类活动材料，促进幼儿搭建、绘画、简单手工制作等精细动作发育。
　　（3）鼓励幼儿自己用水杯喝水、用勺吃饭、协助收纳等。
　　（三）指导建议。
　　1.在各个生活环节中，创造丰富的身体活动环境，确保活动环境和材料安全、卫生。
　　2.充分利用日光、空气和水等自然条件，进行身体锻炼，保证充足的户外活动时间。
　　3.安排类型丰富的活动和游戏，并保证每日有适宜强度、频次的大运动活动。做好运动中的观察及照护，避免发生伤害。
　　4.关注患病婴幼儿。处于急慢性疾病恢复期的婴幼儿，及时调整活动强度和时间；发现运动发育迟缓婴幼儿，给予针对性指导，及时转介。

　　五、语言
　　（一）目标。
　　1.对声音和语言感兴趣，学会正确发音；
　　2.学会倾听和理解语言，逐步掌握词汇和简单的句子；
　　3.学会运用语言进行交流，表达自己的需求；
　　4.愿意听故事、看图书，初步发展早期阅读的兴趣和习惯。
　　（二）保育要点。
　　1.　7-12个月
　　（1）经常和婴儿说话，引导其对发音产生兴趣，模仿和学习简单的发音。
　　（2）向婴儿复述生活中常见物品和动作，帮助其逐渐理解简单的词汇。
　　（3）引导婴儿使用简单的声音、表情、动作、语言表达自己的需求。
　　（4）为婴儿选择合适的图画书，朗读简单的故事或儿歌。
　　2.　13-24个月
　　（1）培养幼儿正确发音，逐步将语言与实物或动作建立联系。
　　（2）鼓励幼儿模仿和学习使用词语或短句表达自己的需求。
　　（3）引导幼儿学会倾听并乐意执行简单的语言指令，积极使用语言进行交流。
　　（4）提供机会让幼儿多读绘本、多听故事、学念儿歌。
　　3.　25-36个月
　　（1）指导幼儿正确地运用词语说出简单的句子。
　　（2）鼓励幼儿用语言表达自己的需求和感受。
　　（3）创造条件和机会，使幼儿多听、多看、多说、多问、多想，谈论生活中的所见所闻。
　　（4）培养幼儿阅读的兴趣和能力，学讲故事、学念儿歌。
　　（三）指导建议。
　　1.创设丰富和应答的语言环境，提供正确的语言示范，保持与婴幼儿的交流与沟通，引导其倾听、理解和模仿语言。
　　2.为不同月龄婴幼儿提供和阅读适合的儿歌、故事和图画书，培养早期阅读兴趣和习惯。
　　3.关注语言发展迟缓的婴幼儿，并给予个别指导。

　　六、认知
　　（一）目标。
　　1.充分运用各种感官探索周围环境，有好奇心和探索欲；
　　2.逐步发展注意、观察、记忆、思维等认知能力；
　　3.学会想办法解决问题，有初步的想象力和创造力。
　　（二）保育要点。
　　1. 7-12个月
　　（1）提供有利于视、听、触摸等材料，激发婴儿的观察兴趣。
　　（2）鼓励婴儿调动各种感官，感知物体的大小、形状、颜色、材质等。
　　（3）引导婴儿观察周围的事物，模仿所看到的某些事物的声音和动作。
　　2. 13-24个月
　　（1）引导幼儿运用各种感官探索周围环境，逐步发展注意、记忆、思维等认知能力。
　　（2）鼓励幼儿辨别生活中常见物体的大小、形状、颜色、软硬、冷热等明显特征。
　　（3）鼓励幼儿在操作、摆弄、模仿等活动中想办法解决问题。
　　3. 25-36个月
　　（1）引导幼儿运用各种感官反复持续探索周围环境，逐步巩固和加深对周围事物的认识。
　　（2）启发幼儿观察辨别生活中常见物体的特征和用途，进行简单的分类，并感受生活中的数学。
　　（3）培养幼儿在感兴趣的事情上能够保持一定的专注力。
　　（4）通过各种游戏和活动，鼓励幼儿主动思考、积极提问并大胆猜想，激发幼儿的想象力和创造力。
　　（三）指导建议。
　　1.创设环境，促进婴幼儿通过视、听、触摸等多种感觉活动与环境充分互动，丰富认识和记忆经验。
　　2.保护婴幼儿对周围事物的好奇心和求知欲，耐心回应婴幼儿的问题，鼓励自己寻找答案。
　　3.在确保安全健康的前提下，支持和鼓励婴幼儿的主动探索。

　　七、情感与社会性
　　（一）目标。
　　1.有安全感，能够理解和表达情绪；
　　2.有初步的自我意识，逐步发展情绪和行为的自我控制；
　　3.与成人和同伴积极互动，发展初步的社会交往能力。
　　（二）保育要点。
　　1. 7-12个月
　　（1）观察了解不同月龄婴儿的需要，把握其情绪变化，尊重和满足其爱抚、亲近、搂抱等情感需求。
　　（2）引导婴儿理解和辨别高兴、喜欢、生气等不同情绪。
　　（3）敏感察觉婴儿情绪变化，理解其情感需求并及时回应。
　　（4）创设温暖、愉快的情绪氛围，促进婴儿交往的积极性。
　　2. 13-24个月
　　（1）引导幼儿用表情、动作、语言等方式表达自己的情绪。
　　（2）培养幼儿愉快的情绪，及时肯定和鼓励幼儿适宜的态度和行为。
　　（3）拓展交往范围，引导幼儿认识他人不同的想法和情绪。
　　（4）引导幼儿理解并遵守简单的规则。
　　3. 25-36个月
　　（1）谈论日常生活中幼儿感兴趣的人和事，引导其通过语言和行为等方式表达情绪情感。
　　（2）鼓励幼儿进行情绪控制的尝试，指导其学会简单的情绪调节策略。
　　（3）创设人际交往的机会和条件，使幼儿感受与人交往的愉悦。
　　（4）帮助幼儿理解和遵守简单的规则，初步学习分享、轮流、等待、协商，尝试解决同伴冲突。
　　（三）指导建议。
　　1.观察了解每个婴幼儿独特的沟通方式和情绪表达特点，正确判断其需求，并给予及时、恰当的回应。
　　2.与婴幼儿建立信任和稳定的情感联结，使其有安全感。
　　3.建立一日生活和活动常规，开展规则游戏，帮助婴幼儿理解和遵守规则，逐步发展规则意识，适应集体生活。
　　4.创造机会，支持婴幼儿与同伴和成人的交流互动，体验交往的乐趣。

　第三章　组织与实施

　　一、托育机构是实施保育的场所，应当提供健康、安全、丰富的生活和活动环境，配置符合婴幼儿月龄特点的家具、用具、玩具、图书、游戏材料和安全防护措施，并根据场地条件合理确定收托规模，配备符合要求的保育人员。

　　二、托育机构负责人负责保育的组织与管理，指导、检查和评估保育人员的工作。

　　三、托育机构保育人员是保育工作的主要实施者，应当具有良好的职业道德和业务能力，身心健康。负责婴幼儿日常生活照料和活动组织，主动了解和满足婴幼儿不同的发展需求，平等对待每一个婴幼儿，呵护婴幼儿健康成长。

　　四、保育工作应当根据婴幼儿身心发展特点和规律，制订科学的保育方案，合理安排婴幼儿饮食、饮水、如厕、盥洗、睡眠、游戏等一日生活和活动，支持婴幼儿主动探索、操作体验、互动交流和表达表现，丰富婴幼儿的直接经验。

　　五、托育机构应当建立信息管理、健康管理、疾病防控和安全防护监控制度，制定安全防护、传染病防控等应急预案，切实做好室内外环境卫生，注意防范和避免伤害，确保婴幼儿的安全和健康。

　　六、托育机构应当与家庭、社区密切合作，充分整合各方资源支持托育机构保育工作，向家庭、社区宣传科学的育儿理念和方法，提供照护服务和指导服务，帮助家庭增强科学育儿能力。

© 北大法宝：（[www.pkulaw.com](https://www.pkulaw.com/)) 专业提供法律信息、法学知识和法律软件领域各类解决方案。北大法宝为您提供丰富的参考资料，正式引用法规条文时请与标准文本核对。
欢迎查看所有[产品和服务](https://www.pkulaw.com/helps/2.html)。法宝快讯：[如何快速找到您需要的检索结果？](https://www.pkulaw.com/helps/70.html)[法宝](https://www.pkulaw.com/helps/69.html)V6有何新特色？

扫描二维码阅读原文

**原文链接：**<https://www.pkulaw.com/chl/9c0e16b7aff8f0e6bdfb.html>
